# The effects of hospice-shared care for gastric cancer patients

**DOI:** 10.1371/journal.pone.0171365

**Published:** 2017-02-03

**Authors:** Kun-Siang Huang, Shih-Ho Wang, Seng-Kee Chuah, Kun-Ming Rau, Yu-Hung Lin, Meng-Che Hsieh, Li-Hsueh Shih, Yen-Hao Chen

**Affiliations:** 1 Department of Family Medicine, Kaohsiung Chang Gung Memorial Hospital and Chang Gung University College of Medicine, Kaohsiung, Taiwan; 2 Division of General surgery, Department of Surgery, Kaohsiung Chang Gung Memorial Hospital and Chang Gung University College of Medicine, Kaohsiung, Taiwan; 3 Gastric cancer team in Kaohsiung Chang Gung Memorial Hospital, Kaohsiung, Taiwan; 4 Division of Hepatogastroenterology, Department of Internal Medicine, Kaohsiung Chang Gung Memorial Hospital and Chang Gung University College of Medicine, Kaohsiung, Taiwan; 5 Division of Hematology-Oncology, Department of Internal Medicine, Kaohsiung Chang Gung Memorial Hospital and Chang Gung University College of Medicine, Kaohsiung, Taiwan; 6 Department of Nursing, Kaohsiung Chang Gung Memorial Hospital, Kaohsiung, Taiwan; 7 Hospice care team in Kaohsiung Chang Gung Memorial Hospital, Kaohsiung, Taiwan; Western General Hospital, UNITED KINGDOM

## Abstract

**Background:**

Hospice care has been proved to result in changes to the medical behaviors of terminally ill patients. The aim of this study was to evaluate the effects and medical behavior changes of hospice-shared care intervention among terminally ill gastric cancer patients.

**Methods:**

A total of 174 patients who died of gastric cancer between 2012 and 2014 were identified. These patients were divided into two groups: a hospice-shared care group (n = 93) and a control group (n = 81).

**Results:**

Among the 174 patients, 84% had advanced stage (stage III or stage IV) cancer. The females and the patients cared by medical oncologists had a higher percentage of hospice-shared care than the males (71% vs 44%, p = 0.001) and those cared by other physicians (63% vs 41%, p = 0.004). Compared to the control group, the hospice-shared care group underwent lower incidence of life sustaining or aggressive medical treatments, including intensive care unit admission (2% vs 26%, p<0.001), intubation (1% vs 27%, p<0.001), cardiopulmonary-cerebral resuscitation (0% vs 11%, p = 0.001), ventilator use (1% vs 27%, p<0.001), inotropic agent use (8% vs 46%, p<0.001), total or partial parenteral nutrition use (38% vs. 58%, p = 0.029), and blood transfusion (45% vs 74%, p<0.001). Besides, the hospice-shared care group had a higher percentage of palliative treatments than the control group, including signed Do-Not-Resuscitate (DNR) orders (95% vs 37%, p<0.001), receiving home hospice care (16% vs 1%, p<0.001), and indicating home as the realistically preferred place of death (41% vs 19%, p = 0.001). The hospice ward admission rate in the hospice-shared care group increased from 30% to 53% from 2012 to 2014.

**Conclusion:**

The use of hospice-shared care for gastric cancer patients could increase the rate of signed DNR orders, decrease the use of life sustaining and aggressive/palliative treatments, and improve quality of life.

## Introduction

Gastric cancer is one of the most frequently occurring malignancies worldwide, with the highest incidences occurring among East Asian populations [1]. In Taiwan, gastric cancer was the 7^th^ leading cause of death in 2014, with a mortality rate of 10 in 100,000 people [2]. Despite the significant improvements that have been made in both surgical technology and chemoradiation therapy, the prognosis of gastric cancer patients remains unsatisfactory. In 2012, advanced gastric cancer (which includes both stage III and stage IV) accounted for about 50% of all gastric cancer patients in Taiwan, with the poorly differentiated type and signet-ring cell type being the types most seen in such advanced cases. In addition, the 5-year-survival rate for gastric cancer from 2007 to 2011 was only 36.3% in Taiwan.

The main treatment for the late stages of gastric cancer remains chemotherapy [3]. However, due to the poor response rate to palliative chemotherapy and the multiple complications that may occur with such therapy or due to the cancer itself (such as gastric outlet/intestinal obstruction, peritoneal carcinomatosis, gastrointestinal bleeding, nausea/vomiting, neutropenia, etc.), the quality of life for advanced gastric cancer patients is generally poorer than that for patients with other malignancies [4]. Moreover, dissatisfaction with the futility and indignity of expensive and intrusive medical treatments used to treat terminally ill cancer patients in Taiwan has previously been recognized [5]. Relatedly, the hospice movement has gradually been adopted in Taiwan since 1983 [6], and hospice utilization was further facilitated in 2000 by Taiwan’s new National Health Insurance [7]. Hospice care is general term covering hospice wards, hospice-shared care, and hospice home care. Hospice-shared care is a care model which was provided by Taiwan National Health Insurance Administration [8]. The hospice-shared care consists of multidiscipline palliative care specialties consultations including physicians, nurses, social workers and religious workers. The hospice-shared care serves for the terminally-ill patients who were admitted and provides the biological, social, psychological and spiritual support without leaving their original care team and environment [9, 10]. Some past studies have indicated that hospice care can result in changes in medical behaviors. Tse et al found that among patients receiving hospice-shared care, there was less use of intensive care units (ICUs) and fewer admissions to acute care wards [11]. Some studies have also reported lower rates of invasive medical interventions such as cardiopulmonary resuscitation (CPR) [11–13] and intubation and mechanical ventilation support [12, 13] being applied to hospice-shared care patients. Furthermore, lower costs of palliative care have been reported for such patients [14].

However, to the best of our knowledge, no previous study has investigated the differences, if any, in the medical behaviors applied to gastric cancer patients receiving hospice-shared care versus those who do not. The aim of this study, then, was to examine whether hospice-shared care influences the medical behaviors of gastric cancer patients.

## Materials and methods

### Patient selection

This study utilized a retrospective design; patients who had been diagnosed with gastric cancer and died between 2012 and 2014 in Kaohsiung Chang-Gung Memorial Hospital were selected. Finally, a total of 174 patients were included, and these patients were divided in to two groups: a hospice-shared care group consisting of 93 patients and a control group of 81 patients. To understand the differences between the hospice-shared care group and the control group, the following life sustaining treatment parameters were examined: 1) the rates of intensive care unit (ICU) admissions; 2) the rates of intubation usage; 3)the rates cardiopulmonary-cerebral resuscitation (CPCR); 4) the rate of ventilator usage and 5) the rates of inotropic agent usage. We also analyzed the following aggressive and palliative medical treatment parameters between these two groups: 1) undergoing chemotherapy one month before death; 2) the rates of total parenteral nutrition (TPN) or partial parenteral nutrition (PPN) usage; 3) the rates at which blood transfusions were provided; 4) the rates of signed Do-Not-Resuscitate (DNR) orders; 5) the rates of home hospice care usage and 6) the rates at which home was indicated as the realistically preferred place of death. We reviewed each patient’s medical records to determine the patient’s hospice-shared care status and the other parameters.

### Statistical analysis

Statistical analyses were performed using the SPSS 17 software package. The chi-square test was used to compare data between patients receiving hospice-shared care and those who did not. For all analyses, two-sided tests of significance were used, with P < 0.05 considered significant.

### Ethics statement

This retrospective analysis was approved by the Chang Gung Medical Foundation Institutional Review Board (201600980B0). Written informed consent of the patients or their family was not judged necessary for this kind of retrospective study by the Chang Gung Medical Foundation Institutional Review Board.

## Results

We identified 174 patients who died of gastric cancer between January 1, 2012, and December 31, 2014, in Kaohsiung Chang Gang Memorial Hospital. The characteristics of those patients are listed in Table 1. The median age was 68 years old. A total of 115 (66%) patients were men, and 59 (34%) were women. Together, the stage III and IV gastric cancer patients accounted for 84% of all the patients (stage III: 33%; stage IV: 51%). Ninety-three (53%) of the 174 patients received hospice-shared care whereas eighty-one (47%) did not. The highest percentage of the primary care physician was medical oncologist (56%) and the second one was general surgeon (22%).

**Table 1:** Characteristics of 174 gastric cancer patients.

Characteristic	Numbers (%)
Age	
<70	91 (52%)
≥70	83 (48%)
Sex	
Male	115 (66%)
Female	59 (34%)
Stage	
I	10 (6%)
II	17 (10%)
III	57 (33%)
IV	90 (51%)
Primary care physician	
Medical oncologist	98 (56%)
General surgeon	38 (22%)
Gastroenterologist	18 (10%)
Others	20 (12%)
Hospice enrollment	
Yes	93 (53%)
No	81 (47%)

The comparison of gastric cancer patients receiving hospice-shared care or not was listed in Table 2. The age distribution had no significant difference between these two groups. In patients receiving hospice-shared care, the females were more than the males with statistically significance (71% vs 44%, p = 0.001). Of the primary care physician, the medical oncology had a higher ratio of hospice-shared care than other specialties (63% vs 41%, p = 0.004).

**Table 2:** Comparison of gastric cancer patients receiving and not receiving hospice care and those who did not.

Variable	Hospice enrollment (N = 93)	No hospice care (N = 81)	P value
Age			0.103
<70	54 (59%)	37 (41%)	
≥70	39 (47%)	44 (53%)	
Sex			0.001
Male	51 (44%)	64(56%)	
Female	42 (71%)	17 (29%)	
Primary care physician			0.004
Medical oncologist	62 (63%)	36 (37%)	
Others	31 (41%)	44 (59%)	

A comparison of the life sustaining treatment of gastric patients who underwent hospice-shared care or not is shown in Table 3. The patients receiving hospice-shared care underwent significantly lower rates of life sustaining interventions, including ICU admission (2% vs 26%, p<0.001), intubation (1% vs 27%, p<0.001), CPCR (0% vs 11%, p = 0.001), ventilator use (1% vs 27%, p<0.001), inotropic agent use (8% vs 46%, p<0.001) than the control group patients.

**Table 3:** Comparison of life sustaining treatment in gastric cancer patients receiving hospice care or not.

Variable	Hospice-care group (N = 93)	Control group (N = 81)	P value
ICU admission	2 (2%)	21 (26%)	<0.001*
Intubation	1 (1%)	22 (27%)	<0.001*
CPCR	0 (0%)	9 (11%)	0.001*
Ventilator use	1 (1%)	22 (27%)	<0.001*
Inotropic agent use	7 (8%)	37 (46%)	<0.001*

DNR: Do-Not-Resuscitaate; ICU: Intensive Care Unit; CPCR: Cardiopulmonary cerebral resuscitation.

*Statistically significant.

The comparison of aggressive and palliative medical treatment of hospice-shared care group and control group was shown in Table 4. The patients receiving hospice-shared care had significantly lower rate of aggressive medical treatments, including TPN/PPN usage (38% vs 58%, p = 0.029), blood transfusion (45% vs 74%, p<0.001) and undergoing chemotherapy one month before death (9% vs 28%, p<0.001) than those of not receiving hospice-shared care. The percentage of signed DNR orders (95% vs 37%, p<0.001), the home hospice care rate (16% vs 1%, p<0.001) and home as realistically preferred place of death rate (41% vs 19%, p = 0.001) were also higher in the hospice-shared care group than the patients without hospice-shared care.

**Table 4:** Comparison of aggressive/palliative medical treatment in gastric cancer patients receiving hospice care or not.

Variable	Hospice-care group (N = 93)	Control group (N = 81)	P value
Chemotherapy use	8 (9%)	23 (28%)	<0.001*
TPN/PPN use	35 (38%)	43 (53%)	0.029*
Blood transfusion	42 (45%)	60 (74%)	<0.001*
Home hospice care	15 (16%)	1 (1%)	<0.001*
Home as realistically preferred place of death	38 (41%)	15 (19%)	0.001*

TPN: Total parenteral nutrition; PPN: peripheral parenteral nutrition.

*Statistically significant.

The changes in the aggressive medical treatments among 174 gastric cancer patients between 2012 and 2014 were listed in Table 5. There was a declining trend of aggressive medical treatments from 2012 to 2014, including ICU admission (17% vs 13% vs 8%, p = 0.412), intubation (17% vs 11% vs 9%, p = 0.492), ventilator use (17% vs 11% vs 9%, p = 0.492), and inotropic agent use (32% vs 23% vs 17%, p = 0.155) although no statistically significances were found. The CPCR rate (3% vs 5% vs 8%, p = 0.392) increased from 2012 to 2014 without statistically significance.

**Table 5:** Changes in the rate of hospice utilization and medical treatment among 174 gastric cancer patients between 2012 and 2014.

Variate	2012 (N = 71)	2013 (N = 56)	2014 (N = 47)	P value
ICU admission	12 (17%)	7 (13%)	4 (8%)	0.412
Intubation	12 (17%)	6 (11%)	5 (9%)	0.492
Ventilator use	12 (17%)	6 (11%)	5 (9%)	0.492
Inotropic agent use	23 (32%)	13 (23%)	8 (17%)	0.155
CPCR	2 (3%)	3 (5%)	4 (8%)	0.392

DNR: Do-Not-Resuscitaate; ICU: Intensive Care Unit; CPCR: Cardiopulmonary cerebral resuscitation.

**Fig 1:**
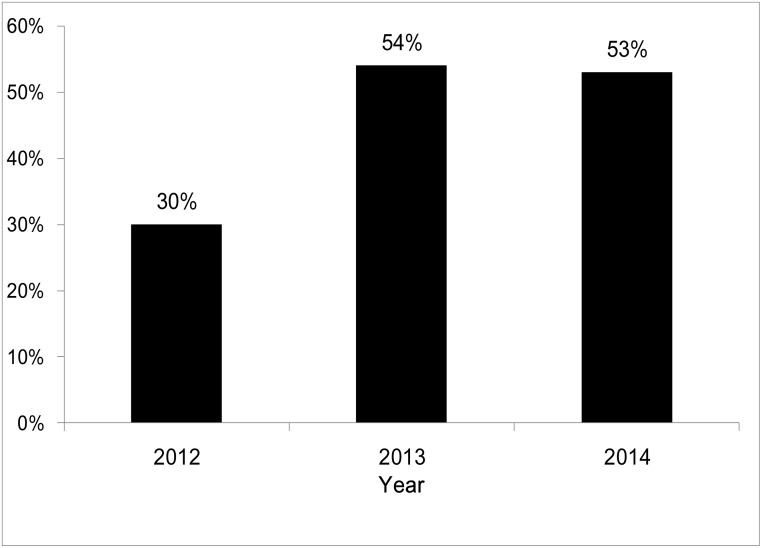
The hospice ward admission rates of the gastric cancer patients who received hospice care.

We also analyzed the annual hospice ward admission rates of the patients who received hospice-shared care, and those results are shown in Fig 1. Among the patients receiving hospice-shared care, the hospice ward admission rate was 30% in 2012. The rate increased to 54% in 2013, and then was 53% in 2014. There was a clearly increasing trend of need and utilization of hospice ward among the patients receiving hospice-shared care.

## Discussion

The prognoses of gastric cancer patients can vary substantially. Patients with the early stages of gastric cancer have been reported to have relatively good outcomes, with five-year survival rates of treated early gastric cancer in modern times being over 90% [15–18]. However, the prognosis of advanced stage gastric cancer remains poor in general. In Taiwan, the 5-year-survival rate for advanced gastric cancer was only about 36% in 2007–2011. In this study, patients with advanced stage gastric cancer accounted for 84% of all the patients. The complications of gastric cancer, including intestinal obstruction, peritoneal carcinomatosis, and gastrointestinal bleeding severely influence quality of life, distressing both patients and their family members [19]. In addition, the medical expenses for advanced stage gastric cancer patients are generally high due to the need for medications and costs associated with various complications [20, 21]. Medical futility in the treatment of advanced stage gastric cancer patients is also an important issue.

There are many factors such as sex, age and primary care physician which may influence the patients enrolling hospice care. From our results, in gastric patients receiving hospice-shared care, the proportion of female was higher than male. The gender difference of patients receiving hospice care was also observed in some studies. Obermeyer et al reported that the female was associated with hospice enrollment in poor-prognosis cancer patients [22]. Another study showed that there were more men than women in patients receiving palliative care consultation service [23]. Until now, there were no consistent findings in gender distribution due to very limited studies. In our study, women seemed to be prone to receive hospice-shared care than men; this may be related to the Chinese culture.

The primary care physician also plays an important role in hospice enrollment in terminally ill patients. From our results, the medical oncologists accounted for the most proportion of primary care physician in patients receiving hospice-shared care. The same finding is also mentioned by some past studies, indicating that patients cared by medical oncologists were significantly more likely to enroll in hospice than patients cared by other specialties. [22, 23] Obermeyer et al assumed that the medical oncologists may make better prognostic estimates and may be more effective in discussing hospice care with patients when compared with other specialties [22].

The utilization of hospice care can reduce hospital stays and the application of invasive medical behaviors, in addition to lowering overall medical costs [24, 25]. Hospice-shared care has always been found to have a positive effect on the utilization of hospice care, the rate of signed DNR orders, and the quality of end-of-life care for terminal cancer patients [26]. In advanced gastric cancer patients, less aggressive chemotherapy may result in a lower rate of ICU admission and longer patient survival than are seen among those patients who continue to receive aggressive chemotherapy [4]. From our results, we found that the use of hospice-shared care for gastric cancer patients significantly and effectively reduced the life sustaining and aggressive medical treatments, including CPCR, intubation, mechanical ventilator use, ICU admission, inotropic agent use, blood transfusion, parenteral nutrition and chemotherapy use, results which were consistent with those of prior studies [4, 24, 25]. The proportions of life sustaining medical treatments including ICU admission, intubation, ventilator use and inotropic agent use declined from 2012 to 2014 although no statistical significance was achieved. This achievement may be contributed to the promotion and education about hospice care for primary care physician by hospice-shared care team. The CPCR rate had a non-statistically significant inclining trend year by year. However, the CPCR events were very rare in our study and which may contribute to bias. By reducing the levels of aggressive medical behaviors, the hospice-shared care may also reduce unnecessary medical expenses and avoid medical futility. In our study, the hospice-shared care group also had a higher rate of signed DNR order, greater home hospice care utilization, and a higher rate of indicating home as the realistically preferred place of death. All of these results indicated that hospice-shared care may improve the quality of life in gastric cancer patients.

In our study, the parenteral nutrition (total PPN/TPN) use rate among all the gastric cancer patients was about 45%. This may have been attributable to the inevitable complications of the cancer, such as intestinal obstruction that would impair the enteral nutrition and cause cancer cachexia [27], resulting in increased morbidity and mortality [28]. When enteral nutrition was impaired by a tumor, parenteral nutrition seemed to be an alternative means of supplying nutrition. Torelli, Campos et al indicated, however, that TPN use in terminal ill cancer patients did not improve the quality of life nor change the ultimate outcome [29]. Decreasing the use of parenteral nutrition may avoid unfavorable complications such as infection, while also reducing medical expenses. However, Rosania et al concluded that short-term complementary home parenteral nutrition in advanced gastric cancer is associated with an improvement in quality of life, nutrition status, and functional status [30]. In any event, because the effects of parenteral nutrition in advanced gastric cancer patients remain controversial, detailed and individualized evaluations for parenteral nutrition may be needed for gastric cancer patients. In Chinese culture, oral intake and nutrition supply play an important role in family relations, especially for severely ill patients. Most family members would thus like to maintain oral intake or enteral nutrition in terminally ill patients. In this study, however, if oral intake or enteral nutrition was not feasible due to disease progression, parenteral nutrition was usually requested. Therefore, our study revealed that even under such a cultural atmosphere, hospice-shared care could effectively reduce the use of parenteral nutrition in gastric cancer patients compared with the rate at which it was given to the control group, indicating that hospice enrollment really changes patients’ behaviors and insights.

In our study, the hospice ward admission rate in the hospice-shared care group increased (from 30% to 54%) from 2012 to 2013, which may have been attributable to the promotion of the hospice concept by the hospice-shared care providers. However, the hospice ward admission rate did not change a lot from 2013 to 2014. There may have been two reasons for this. First, the quality of the hospice-shared care in the ordinary unit may have increased, such that the patients did not require admission to the hospice ward in order to receive the same hospice care. Second, some patients followed at our outpatient clinic or discharged from our hospital were transferred to home hospice care, and thus required no hospice ward admission. Therefore, the hospice ward admission rate even had a slight decrease (from 54% to 53%) from 2013 to 2014.

Our study had some limitations. First, it was a retrospective study of patients treated in a single institution. Second, the sample size was small. Only 174 patients were included in the study. Thus, unknown sources of bias may exist in the findings. Further large and rigorous studies may thus be needed to examine the validity and reliability of the results presented herein.

## Conclusions

The use of hospice-shared care for gastric cancer patients can increase the rate of signed DNR orders, lower the use of aggressive medical interventions, decrease medical futility, and improve the quality of life. Because of the generally poor prognosis of gastric cancer, especially in the advanced stage, early hospice-shared care is important and necessary for such patients.
